# A new lectotype for *Passiflora laurifolia* L.

**DOI:** 10.3897/phytokeys.95.22324

**Published:** 2018-02-15

**Authors:** Maxime Rome, Geo Coppens d’Eeckenbrugge

**Affiliations:** 1 Université Grenoble Alpes, CNRS, SAJF, F-38000 Grenoble, France; 2 CIRAD, UMR AGAP, Avenue Agropolis, 34398 Montpellier, France; 3 AGAP, Université de Montpellier, CIRAD, INRA, Montpellier SupAgro, Montpellier, France

**Keywords:** Passifloraceae, subgenus *Passiflora*, series *Laurifoliae*

## Abstract

From the "Description des plantes d'Amérique" by Plumier, in 1693, to the "Species Plantarum" by Linnaeus in 1753, several dubious synonymies of *Passiflora laurifolia* L. were proposed, generating a persisting confusion. A revision of the process, which led to the Linnaean description of this species, shows that the type finally chosen by Cusset does not match the original description. A new lectotype for the species and a more complete description are proposed from field observations, herbarium and bibliographic data.

## Introduction

Amongst Passifloraceae Jussieu (1805) ex Roussel (1806), *Passiflora* Linnaeus (1753) is the most important genus with about 576 species, mostly distributed in tropical America (Krosnick et al. 2013). It includes lianas with tendrils, trees and shrubs, with alternate leaves, axillary stipules, extra-floral nectaries on the petiole and/or the surface of the leaves or even bracts and flowers with crowns of filaments and an androgynophore (Ulmer and MacDougal 2004). Five subgenera are currently recognised: *Passiflora*, *Astrophea* (Candolle, 1822) Masters (1872), *Decaloba* (Candolle, 1822) Reichenbach (1828), *Deidamioides* (Harms, 1925) Killip (1938) and *Tetrapathea* (Candolle, 1822) P.S. Green (1972) (Krosnick et al. 2009), each one subdivided into supersections, sections and/or series.

Supersection Laurifolia (Cervi, 1997) Feuillet and MacDougal (2003) is part of subgenus Passiflora. It includes series *Laurifoliae* Killip (1938) ex Cervi (1997), which forms a morphologically very homogenous group, with a very difficult taxonomy. It is composed of 24 species including glabrous to pubescent plants, with stems that are terete to angular and sometimes corky on old parts; leaves that are entire, oblong-lanceolate, not peltate, with entire to glandular-serrulate margins, biglandular petioles; and stipules that are setaceous or linear, early deciduous. Their three bracts, free at the base, with entire or serrulate-glandular margins, are more than 1 cm long. Their flowers are pendent, usually large and showy, often fragrant with a short hypanthium and two campanulate series of long external filaments and a variable number of series of reduced internal filaments (Rome and Coppens d’Eeckenbrugge 2017). They are valued for their soft and sweet pulp and some species are grown commercially, including *P. laurifolia*.

*Passiflora laurifolia* Linnaeus (1753) was one of the earliest passion flowers to be described and, logically, the first one in the series *Laurifoliae*. It was first mentioned and illustrated in the “Description des plantes d'Amérique” by Plumier (1693), amongst 12 passion flower species from the Caribbean, under the polynomial *Clematis indica, fructu citriformis, foliis oblongis* (climbing plant from the Indies, with lemon-shaped fruits and oblong leaves). Linnaeus (1753) recognised Plumier’s polynomial, as well as several other polynomials and illustrations under his *P. laurifolia*; however, as stated by Killip (1938), this species is not represented by a true type in the Linnaean Herbarium and several Linnaean specimens correspond to the very similar *P. nitida* Kunth (1817), another member of the series *Laurifoliae*. Thus, when Cusset (1967) finally typified *P. laurifolia*, he chose, amongst Linnaean materials, an illustration on plate 21 by Merian (1705) representing in fact a specimen of *P. nitida* from Suriname.

Here, the pre-Linnaean and Linnaean treatments of *P. laurifolia* are revised to support the choice of Plumier’s illustration as a new lectotype for this species. Furthermore, the authors compare specimens from the Antilles and South-America to ascertain that they are conspecific and correspond to the successive descriptions of *P. laurifolia*, discarding any geographical reason for the ancient confusion. A range of materials of *P. nitida* is also examined. From these observations, a complete description for *P. laurifolia* is presented.

## Materials and methods

Three specimens from the herbarium of the Linnean Society of London (LINN) were observed: Linnaeus (*P. laurifolia*), not numbered, of unknown origin, number 1070.2 in the herbarium of the London Linnaean Society; Linnaeus 74 (*P. nitida*), from Suriname; and Linnaeus 152, (*Passiflora* sp.), of unknown origin.

Other materials of *P. laurifolia* examined include 28 herbarium specimens from the Antilles and South America, as well as ten living specimens of *P. laurifolia* with flowers and fruits in Martinique and Guadeloupe. Other materials of *P. nitida* examined include 50 herbarium specimens from South America and living plants with flowers and fruits from eight populations in French Guiana. Examined materials are presented in more details in Appendix 1 and Appendix 2.

## Results and discussion

### Pre-Linnaean treatment

**Figure 1.**
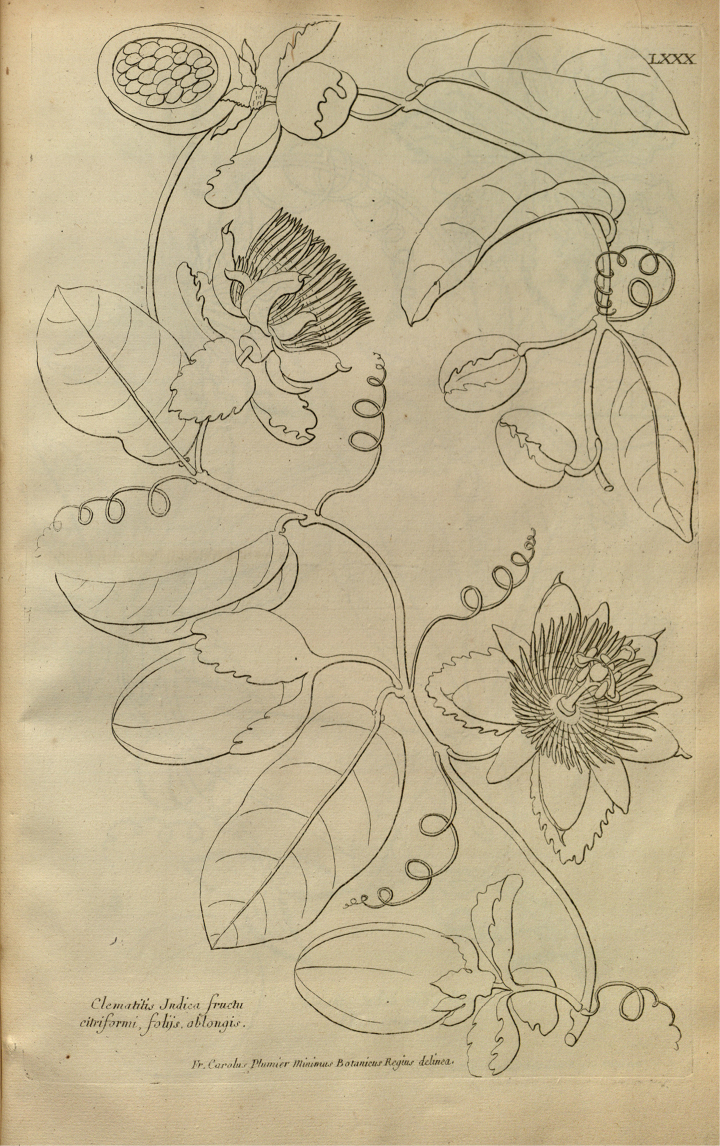
Illustration of *Passiflora laurifolia* in “Description des Plantes d'Amérique” by Plumier (1693).

The authors transcribe here the long and precise description of *P. laurifolia* in French by Plumier (1693) giving the colour and size of each part of the plant. The plant is woody with only one leaf or fragrant flower at each node. The leaves are ovate-elongated, slightly pointed at apex and indented at base, with two glands at the petiole apex. The flowers are enclosed in three green membranaceous bracts [“dans trois feuilles vertes membraneuses”]. The perianth is white, densely dotted with brown red inside. The two outer series of filaments are not equal, the innermost being about 3.75 cm long and the outermost over 1.25 cm long. They are purple on the distal half and striped with red and purple on their proximal half. Inside the flower, there are two other, very short, slender and whitish series of filaments. The androgynophore is yellowish, marbled with red, with three red styles, three yellow stigmas, a yellow ovary and five stamens with whitish anthers. The fruit is the size of a chicken egg, with three longitudinal ribs, turning orange with many tiny dots at maturity. Plumier describes its pericarp as thin, leathery, [“de l'épaisseur et la consistance d'un gros cuir molasse indiquant que l'enveloppe du fruit n'est pas très épaisse”] and pubescent [“écorce cotonnée par dehors”]. The whitish pulp is mucilaginous and sweet and contains black heart-shaped seeds. The species is cultivated in home gardens and the fruits are ripe from April to May. Plumier’s description of *P. laurifolia* was well illustrated by the drawing presented at Figure 1.

In addition, Plumier (1693) pointed out that the fruit is very similar to those of the “murucuia guaçu” (meaning large passion fruit) of Georg Marcgrave in Ray (1686) and the “murucuia guaçu” of Piso (1648), raising the possibility that they could belong to the same species. However, these two species do not have simple leaves and they cannot even match any species description within series *Laurifoliae*.

Plukenet (1696) considered Plumier’s ‘*Clematis indica, fructu citriformis, foliis oblongis*’ a synonym of ‘*Passiflora arborea Laurinis foliis*’ represented in table 211 in the book of Plukenet (1692). Indeed, the latter includes a drawing of a passion flower with oblong leaves and two glands at the petiole apex; however there is no textual description of the species and the absence of flowers in the drawing allows no further identification of the species. Additionally, Plukenet (1696) considered two other synonyms: Marcgrave’s ‘*Murucuja* 4.s. *Pyriformis altera*’ and an illustrated description of 'Quauh Chichic Patli' by Hernández de Toledo (1651). However, while the synonymy of the former cannot be verified because it gives no indication of the position of petiolar glands, the drawing of the latter shows a plant with opposite leaves, which cannot belong to the genus *Passiflora*.

Van Royen (1740) described several species from the Leiden Botanical Garden and its surroundings. Amongst these, *Passiflora laurifolia* sensu Plumier (1693) was renamed ‘*Passiflora foliis solitariis oblongis intergerrimis, floribus solitariis, involucro tripartite dentate*’. Van Royen (1740) added ‘Haec duas habet sub basi glandulas convexas’. In his collection deposited at the National Herbarium of the Netherlands and inventoried by Thijsse and Veldkamp (2003), there is no voucher material of *Passiflora laurifolia*.

**Figure 2.**
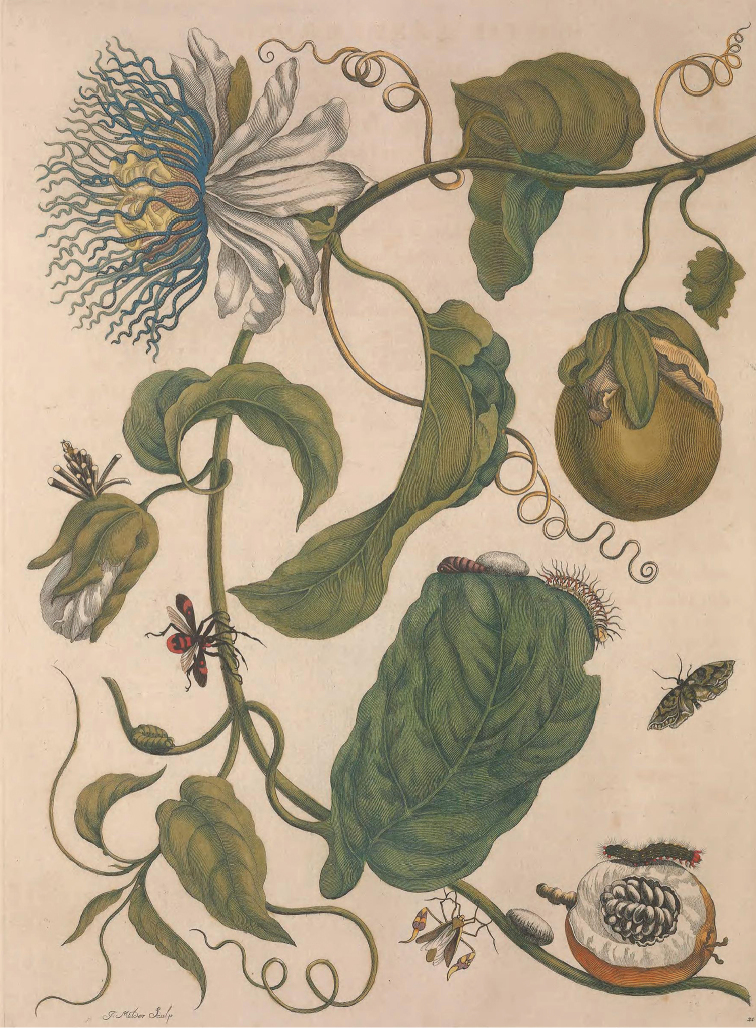
Illustration of the ‘marquiaas’ in “Metamorphosis Insectorum Surinamensium” by Merian (1705).

Linnaeus (1749) named *Passiflora laurifolia* as ‘*Passiflora foliis indivisis integerrimis, involucris dentatis*’ (*Passiflora* with undivided leaves and dentate involucres). Amongst synonyms, he quoted the above-mentioned polynomials of Plumier (1693), Plukenet (1692) and van Royen (1740), as well as ‘*Granadilla fructu citriformi, foliis oblongis*’ of Tournefort (1700) and an illustration by Merian (1705, pl. 21), where plants are drawn with their pollinator and predator insects. In the legend, Merian (1705) named the plant under the vernacular name ‘*marquiaas*’, which is related to the generic vernacular terms *murucuia* and *maracuyá* (cf. Cuvier, 1823), used for all passion fruits across South America. On the drawing by Merian (1705), presented in Figure 2, several diagnostic traits are inconsistent with the description of Plumier (1693): flowers with white perianth (instead of flowers densely dotted with brown red inside) and equal (instead of unequal) outer series of blue corona filaments. The ovoid fruit is uniform yellow or green when unripe (instead of dotted with darker ribs), with a thick mesocarp (instead of a thin one).

Linnaeus (1749) specified that *P. laurifolia* has glabrous and undulate to flat leaves and that bracts are of the same length as the flowers [“*involucrum magnitudine floris*”], which was a new observation in relation to Plumier’s description.

### Linnaean treatment

In *Species Plantarum*, Linnaeus (1753) maintained his description of *P. laurifolia* from *Amoenitates academicae*, with the same synonyms but he did not designate a type of this species. He considered the species as only native to Suriname although he based his species’ description on that of Plumier from Caribbean specimens. Moreover, the white perianth, the two equal outer series of filaments and the uniformly green immature fruit with a thick pericarp, show that Merian’s ‘marquiaas’ belongs to *P. nitida*.

Killip (1938) specified that *P. laurifolia* is not represented by a true type in the Linnaean Herbarium even if the latter includes three specimens identified as belonging to this complex group. Only one of them corresponds unambiguously to *P. laurifolia*; this is Linnaeus nn (no collection number; number 1070.2 in the herbarium of the London Linnaean Society), determined as *P. pallida* by Linnaeus, with a note from the hand of J.E. Smith: “*laurifolia* Jacq. non Linn.”. Thus, Smith indicated that the herbarium specimen does not match the description of *P. laurifolia* by Linnaeus but corresponds to that of Jacquin (1776) in *Hortus Vindobonensis* where this author specifies that *P. laurifolia* has two series of filaments, the outer series being shorter. Below the first annotation, “*pallida*” can be read, hand-written by Linnaeus and next to that name, another annotation from Smith: “non Plumeri icon”). Here, Smith referred to an illustration of Plumier, cited in Linnaeus’ description of *Passiflora pallida* Linnaeus (1753), a species of subgenus Decaloba. This suggests that Linnaeus was not comfortable with this group of plants.

The specimen Linnaeus 152, collected in Suriname, was determined by Linnaeus as “*Passiflora dubia*” (doubtful *Passiflora*) in *Plantae Surinamensis* (1775). In this book, he gave a brief description of the plant that does not allow the determination of the species in the absence of bracts. The specimen Linnaeus 74, from Suriname, also noted as “*Passiflora dubia*” in Linnaeus’ *Plantae Surinamenses*, is in fact *P. nitida* (the fourth inner series of filaments close to the nectary chamber of the flower is easily observable on the specimen), the species described by Kunth in 1817.

### Post-linnean treatment

In his *American species of Passifloraceae*, Killip (1938) simply followed Linnaeus, maintaining the contradiction between the description of *P. laurifolia* taken from Plumier and Merian’s illustration representing *P. nitida*. Later, Cusset (1967) mentioned *P. laurifolia* in Flore du Cambodge du Laos et du Vietnam, considering Merian’s drawing as the type of this species.

### Analysis of herbarium and living specimens

**Figure 3.**
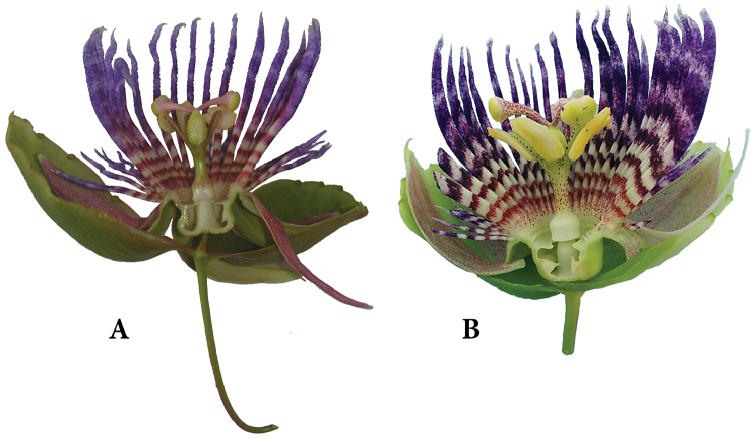
*Passiflora laurifolia*. **A** Flower from Trujillo, Venezuela (photograph courtesy of M. Molinari) **B** Flower from Le Moule, Guadeloupe (photograph courtesy of F. Booms).

The authors’ literature review and observations confirm that *P. laurifolia* is present in all of the Caribbean islands; in fact, it is the only species of series *Laurifoliae* in the Antilles. Regarding its presence on the South American continent, the examined herbarium specimens document its presence in Suriname, Guyana, Venezuela and Brazil. A detailed analysis of the morphology of *P. laurifolia* from the Caribbean and South America shows that the internal structure of the flower is identical in both regions (Figure 3).

A comparison of the successive descriptions of *P. laurifolia* shows that the original one, provided by Plumier (1693) is the most complete and comprehensive and constitutes the common reference linking all subsequent descriptions of the species. Even now, it allows the discrimination of *P. laurifolia* amongst the 24 species composing series *Laurifoliae* (Rome and Coppens d'Eeckenbrugge 2017). The species described by Piso (1648), Hernández de Toledo (1651) and Marcgrave in Ray (1686) are to be invalidated and the corresponding polynomials cannot be considered as earlier synonyms. The descriptions of van Royen (1740) and Plukenet (1696) are too imprecise, their unique interest being that they refer to that of Plumier (1693). Linnaeus’ descriptions (1749, 1753) of *P. laurifolia* were based on the morphology of two different species, the one described by Plumier (1693) and an iconography of a plant that turned out to be *Passiflora nitida*. This confusion has persisted until now, with the treatments of Killip (1938) and Cusset (1967). According to the article 9.19(b) of the International Code of Nomenclature (McNeill et al. 2012), the choice of the lectotype may also be superseded if one can show that it is in serious conflict with the protologue and another element is available that is not in conflict with the protologue. Thus, the iconotype of Merian (1705) is replaced here by that of Plumier (1693). A complete description of *P. laurifolia* is also presented here, based on that of Plumier and examination of materials from the Antilles and the Guianas.

#### Passiflora laurifolia

Taxon classificationPlantaeMalpighialesPassifloraceae

L. Sp. Pl. 956. 1753

##### Lectotype.

Plum. PL. Amer. Pl. 80. 1693, from the Antilles, very probably the Martinique island where Plumier worked. Designated here.

##### Description.

Liana strong. Stem terete, glabrous and green; internodes 3.5–14 cm long. Tendrils cone-shaped, glabrous. Stipules linear, falcate, green yellowish to green brown, eglandular to glandular (0–2 glands), glabrous, 5.5–13.2 × 0.4–1 mm (including an arista, 0–1.8 mm long), deciduous. Petiole 1.1–2.9 cm long, green to dark green, slightly caniculate adaxially, glabrous, with two oval sessile glands situated at petiole apex. Leaves simple, 7.3–14.2 × 4.4–7.5 cm, glabrous throughout, green to dark green, adaxial surface lustrous, rounded to cordate at base, acute (angle within 45 to 90°) at apex, slightly acuminate and mucronate; leaf margin entire or glandular (7–25 marginal nectaries). Peduncles 1.5–7.4 cm long, terete, green, glabrous to slightly pubescent, strong (diameter about 1.3–2.4 mm); pedicel 6.7–15 mm long. Bracts permanent (until fruit maturity), slightly pubescent on both sides, green, concave, 2.8–5.5 cm long, 2.2–4.9 cm wide (same length as flowers), with 4–11 marginal nectariferous sessile glands in distal half. Flowers axillary, pendulous, 22–33 mm long (from the nectary chamber to the ovary apex). Hypanthium slightly pubescent, green outside and white inside, 2.59–5.94 mm, with a diameter of 10.35–16.64 mm at the base of sepals. Nectary chamber slightly pubescent, green outside and white inside, 3.1–5.9 mm long, with a diameter of 8.8–17.9 mm. Sepals glabrous, oblate, 2.9–5.1 cm long, 1–1.7 cm wide, adaxial surface white with a sparse to very dense red punctuation, abaxial surface green with red-brown dots, slightly keel-shaped in distal half with a short awn (1–2 mm long). Petals glabrous, oblate, 2.8–4.9 cm long, 0.7–1.2cm wide, white with a sparse to very dense red punctuation. Corona filaments in 5–7 series, banded white and red to dark purple (purple on the distal half and striped with red to purple on their proximal half); two major series, slightly curved, the outer series 12–30 mm long, the second series 24–43 mm long; others series about 1 mm long. Ovary pubescent, yellow to green, 7–11 mm long; styles, whitish with red purple dots, 8–13 mm long, stigmas light yellow to green. Stamens 7–11 mm long. Androgynophore glabrous, white greenish with red dots, 10–16 mm long with an enlarged base about 10 mm wide. Operculum membranaceous, 0.5–1.1 mm long, recurved, shortly fimbriated at margin. Fruit ovoid, pubescent, 4.7–8.4 cm long, 3.8–6.3 cm in diameter, round to triangular transversal section, epicarp about 0.5–0.9 cm thick; immature fruits green with white dots and with six longitudinal ribs (three of them conspicuous); mature fruits yellowish orange with many tiny light orange dots. Pulp transluscent and sweet. Seeds heart-shaped, black.

## Conclusion

The original description of *P. laurifolia* by Plumier (1693) is the common reference linking all subsequent descriptions of the species. Linnaeus’ descriptions (1749, 1753) involved two iconographies: the one from Plumier (1693) and one from Merian (1705) representing the closely related *Passiflora nitida*, described by Kunth (1817). The latter was mistakenly chosen by Cusset as the lectotype of *P. laurifolia*. To restore consistency, the iconotype of Merian has been replaced by that of Plumier (1693) and a more complete description of *P. laurifolia* has been presented from field observations, herbarium and bibliographic data.

## Supplementary Material

XML Treatment for Passiflora laurifolia

## Appendix 1

**Selected specimens examined**

***Passiflora laurifolia*** (examined on herbarium specimens): **ANTIGUA.** Macarthy Hills, 28 Aug 1938, Box 1524 (MO). **CUBA.** Guantánamo, Rio Navas, Mar 1910, Shafer 4411 (MO). **DOMINICAN REPUBLIC.** Rancho la Cumbre, July 1978, Liogier 27709 (NY). **DOMINICA.** Hamlet of Concord, 0.9 km E of the Pagua River, 14 Aug 1992, Higgins 35 (FTG). **GRENADA.** St Patrick, N of Prospect, 3 Dec 2002, Hawthorne 970 (FHO). **GUADELOUPE.** Petit Bourg, 22 April 1988, Fournet 4368 (P). Basse Terre, 1893, Duss 604 (P), 1894, Duss 3249 (P). **MARTINIQUE.** Carbet, Case Pilote, 1901, Duss 4634 (P); Presqu'île de la Caravelle, 16 Fev 1977, Sastre 5302 (P); Saint Pierre, Le Marin, 1877, Duss 883 (NY). **PUERTO RICO.** Jussieu 16670 (P); Río Piedras, 8 Aug 1979, Woodbury nn (MO). **SAINT EUSTATIUS.** Top of the Quill, 4 June 1906, Boldingh 249 (U). **SAINT LUCIA.** Matout Road to Quilesse, 1 Feb 1985, Howard 20002 (NY). **SAINT VINCENT.** Jan 1890, Eggers 6958 (P). **TRINIDAD.** San Fernando Hill, 18 July 1926, Broadway 6368 (K). **U.S. VIRGIN ISLANDS.** St Croix, Scenic Drive, west slope of Mt. Eagle, 12 Jan 1980, Fosberg 59228 (FTG); St John, Maho Bay Quarter, 23 August 1987, Acevedo-Rodríguez 1924 (NY); Tortola, Sage Mt above Carrot Bay, 9 Feb 1966, D'Arcy 702 (MO); Saint Thomas, nn (P), August 1882, Drake 765 (P). **BRAZIL. Maranhão**: Island of São Luiz, Fe. 1939, Froes 11514 (NY); **Roraima**: SEMA ecological Reserve, Ilha de Maracá, Milliken 49 (INPA). **Unknown origin**: Linnaeus nn, number 1070.2 in the herbarium of the London Linnean Society (LINN). **GUYANA.** Rupununi Distr., N of Shea, 18 Jan 1994, Jansen-Jacobs 3240 (K); Western extremity of Kanaku Mountains, in drainage of Takutu River, 4 March 1938, Smith 3157 (K). **SURINAME.** Mts Bakhuis, 5 April 2006, Bordenave 8382 (CAY), 11 April 2006, Bordenave 8464 (CAY). **VENEZUELA. Bolívar**: Municipio Piar, isla en el Lago de Guri, 23 May 1989, Aymard 7610 (MO); **Anzoátegui**: Ijigua, Headwaters of Rio León, northeast of Bergantín, 27 Feb 1945, Steyermark 61250 (US).

***Passiflora nitida*** (herbarium specimens): **BRAZIL. Amazonas**: Manaus, Rio Negro, Dec 1901, Ule 5974 (K); São Carlos, April 1854, Spruce 3472 (P, K); Street of Aleixo, 27 Dec 1973, Ramos P20141 (K); El Marco, North of Leticia-Tabatinga Road, 24 July 1973, Prance 16843 (NY); Island of Inambú, 19 Nov 1952, Romero-Castañedo 3633 (NY); **Rondônia**: Porto Velho, 30 March 2011, Simon 1280 (CEN). **COLOMBIA. Amazonas**: Santa Isabel, Indigenous Reserve Mirana, 25 May 1984, La Rotta 419 (COAH); Igara Paraná, Milan, 28 Aug 1987, Henao 34 (COAH); about 6 km north of Leticia at Santa Isabella, 28 Feb 1974, Gillett 16526 (COL); Río Igaraparano, 10 June 1942, Schultes 3950 (K); Corregimiento de Tarapacá, 31 Aug 2004, López 8578 (COAH); **Caquetá**: Municipality of Doncello, 21 May 2003, Castaño 1649 (COAH); **Meta**: municipality Vista Hermosa, 6 Jan 2006, Betancur 12050 (COL); **Guaviare**: Municipality San José del Guaviare, 25 Aug 1995, Cárdenas 6535 (COAH); **Vaupés**: Raudal Jirijirimo, Pacoa, 22 Mar 2008, Betancur 13611 (COL); Municipality of Mitú, 22 Mar. 2003, Betancur 10030 (COL). **FRENCH GUIANA.** Indigenous village of Mana, Feb 1856, Sagot nn (P); Crique Petit Laussat, Bassin de la Mana, 9 Feb 1990, Cremers 11336 (CAY) ; Savane Gabrielle, 22 April 1979, Prevost 564 (CAY). **GUYANA.** Konashen area, Essequibo River, 24 Sept 1989, Jansen-Jacobs 1804 (P, K, CAY); Basin of Essequibo River, near mouth of Onoro Creek, 15 Dec 1937, Smith 2820 (K); Mataruki River, upper Essequibo, 4 Dec 1935, Myers 5826 (K); Christianburg, Demerara River, March 1916, Persaud nn (K); North West District, Barama River, Kariako, 10 July 1996, Van Andel 875 (U); Mabura Hill Region, Ekuk compartment, 17 Feb 1993, Ek 734 (U); Potaro River, Kaieteur Plateau, 9 March 1962, Cowan 2080 (US);, Naamryck Canal, 14 April 1989, Gillespie 1017 (US); Mabaruma, 14 July 1934, *Archer 2305* (K); Mabaruma, Aruka River, 7 March 1945, Fanshawe 5108 (K, P); Coast lands, June 1886, Jenman 5406 (K); Rupununi district, Kuyuwini River, 9 Feb. 1991, Jansen-Jacobs 2482 (K, P, U); Potaro-Siparuni Region, Pakaraima Mountains, 24 Oct 1994, Mutchnick 286 (US). **PERU. Loreto**: District of Iquitos, trail Iquitos to San Juan, 7 Feb 1932, Mexia 6488 (K); prov. Mariscal Ramón Castilla, 7 April 2003, Beltrán 5633 (USM); road of Santo Tomás, south of Iquitos, 12 Aug 1972, Croat 19107 (USM); Province of Requena, District of Jenaro Herrera, 17 Feb 2010, Torres LAT331 (USM); Iquitos region, Road to Santa Clara, 14 June 1966, Martin 1012 (K); Maynas, department of Iquitos, Road of Santo Tomás, 11 Feb 1983, Rimachi 6559 (USM); Department of Iquitos, Rio Momón, 30 Jan 1985, Rimachi 7714 (USM); Iquitos, near Versalles, 25 Feb 1969, Plowman 2567 (USM); Prov. Maynas, Rio Nanay, 6 sept 1974, Foster 4066 (F); **Madre de Dios**: Rio Manú, Pakitza station, 20 Nov 1980, Foster 5779 (USM); Manú National Park, 15 Aug 1983, Gentry 43605 (MO); along banks of Río La Torre, 27 Jan 1989, Smith 1637 (USM); **Pasco**: Province of Oxapampa, District of Palcazu, Iscozacín, 16 Nov 2007, Rodríguez 33 (MO); **Cuzco**: Quispicanchis, Limonchayoc, 25 April 1984, Knapp 6404 (USM); Quispicanchis, along trail 25 km SW of Quincemil, 8 Oct 1976, Wasshausen 739 (USM). **SURINAME.** Linnaeus 74 (LINN). Forest of the Station, Groningen, 10 May 1916, Samuels 124 (K). **VENEZUELA. Amazonas** : Río Orinoco, along river just below mouth of Yapacana cano, 18 June 1959, Wurdack 43025 (K). Wankéhe, Cano Marneto, Lower Ventuari, 30 July 1976, Lister 606 (K).

***Passiflora nitida*** (observations on living materials): **FRENCH GUIANA.** Road of Apatou, 26 Nov 2009, Rome 234 (LYJB); Road of Cacao, May 2008, Rome 67 (LYJB); Road of Cacao, 25 Nov 2009, Rome 207 (LYJB); Escol, May 2008, Rome 17 (LYJB); Road of St Georges, May 2008, Rome 49 (LYJB), Rome 130 (LYJB), Rome 139 (LYJB); Road of Tonnegrande, Dec 2009, Rome 203 (LYJB).

***Passiflora* sp.**: Linnaeus 152 (LINN).

## Appendix 2

**Index to numbered collections**

Acevedo-Rodríguez, P. 1924 (laurifolia).

Andel (van), T. 875 (nitida).

Archer, W. A. 2305 (nitida).

Arcy (D’), W. G. 702 (laurifolia).

Aymard, G. 7610 (laurifolia).

Beltrán, H. 5633 (nitida).

Betancur, B. 10030, 12050, 13611 (nitida).

Boldingh, I. 249 (laurifolia).

Bordenave, B. 8382, 8464 (laurifolia).

Box, H.E. 1524 (laurifolia).

Broadway, W. E. 6368 (laurifolia).

Cárdenas, D. 6535 (nitida).

Castaño, N. 1649 (nitida).

Cowan, R.S. 2080 (nitida).

Cremers, G. 11336 (nitida).

Croat, T. 19107 (nitida).

Duss, A. 604, 883, 3249, 4634 (laurifolia).

Drake, E. 765 (laurifolia).

Eggers (von), H.F. A. 6958 (laurifolia).

Ek, R.C. 734 (nitida).

Fanshawe, D.B. 5108 (nitida).

Fournet, A. 4368 (laurifolia).

Fosberg, F.R. 59228 (laurifolia).

Foster, R.B. 4066, 5779 (nitida).

Froes, R. de Lemos 11514 (laurifolia).

Gentry, A. 43605 (nitida).

Gillett, J.M. 16526 (nitida).

Gillespie, L.J. 1017 (nitida).

Hawthorne, W. 970 (laurifolia).

Henao, C.I. 34 (nitida).

Higgins, J. 35 (laurifolia).

Howard, R.A. 20002 (laurifolia).

Jansen-Jacobs, M.J. 3240 (laurifolia); 1804, 2482 (nitida).

Jenman, G. S. 5406 (nitida).

Jussieu (de), A. 16670 (laurifolia).

Knapp, S. 6404 (nitida).

La Rotta, C. 419 (nitida).

Linnaeus, C. nn (laurifolia) ; 74 (nitida) ; 152 (sp.).

Liogier, A.H. 27709 (laurifolia).

Lister, J.R.A. 606 (nitida).

López, R. 8578 (nitida).

Martin, R.T. 1012 (nitida).

Mexia, Y. 6488 (nitida).

Milliken, W. 49 (laurifolia).

Mutchnick, P. 286 (nitida).

Myers, J.G. 5826 (nitida).

Persaud, N. nn (nitida).

Plowman, T. 2567 (nitida).

Prance, G.T. 16843 (nitida).

Prevost, M.F. 564 (nitida).

Ramos, J.F. 20141 (nitida).

Rimachi, Y. 6559, 7714 (nitida).

Rodríguez, D. 33 (nitida).

Rome, M. 17, 49, 67, 130, 139, 203, 207, 234 (nitida).

Romero-Castañedo, R. 3633 (nitida).

Sagot, P.A. nn (nitida).

Samuels, J.A. 124 (nitida).

Sastre, C. 5302 (laurifolia).

Schultes, R. E. 3950 (nitida).

Shafer, J.A. 4411 (laurifolia).

Simon, M.F. 1280 (nitida).

Smith, S.F. 1637, 2820 (nitida).

Spruce, R. 3472 (nitida).

Steyermark, J.A. 61250 (laurifolia).

Torres, L. 331 (nitida).

Ule, E.H. G. 5974 (nitida).

Wasshausen, D.C. 739 (nitida).

Woodbury, R.O. nn (laurifolia).

Wurdack 43025 (nitida).
